# Cathepsin K Is Present in Invasive Oral Tongue Squamous Cell Carcinoma *In Vivo* and *In Vitro*

**DOI:** 10.1371/journal.pone.0070925

**Published:** 2013-08-07

**Authors:** Carolina C. Bitu, Joonas H. Kauppila, Andréia Bufalino, Sini Nurmenniemi, Susanna Teppo, Meeri Keinänen, Suvi-Tuuli Vilen, Petri Lehenkari, Pia Nyberg, Ricardo D. Coletta, Tuula Salo

**Affiliations:** 1 Department of Diagnostics and Oral Medicine, Institute of Dentistry, University of Oulu, Oulu, Finland; 2 Department of Pathology, Institute of Diagnostics, University of Oulu, Oulu, Finland; 3 Department of Surgery, Institute of Clinical Medicine, University of Oulu, Oulu, Finland; 4 Department of Cell Biology of Oral Diseases, Institute of Dentistry, Biomedicum Helsinki Faculty of Medicine, University of Helsinki, Helsinki, Finland; 5 Department of Anatomy and Cell Biology, University of Oulu, Oulu, Finland; 6 Oulu University Hospital, Oulu, Finland; 7 Piracicaba Dental School – UNICAMP, University of Campinas, Piracicaba, São Paulo, Brazil; Stanford University, United States of America

## Abstract

**Objectives:**

Cathepsin K, a lysosomal cysteine protease, is expressed in the tumor microenvironment (TME) of skin carcinoma, but nothing is known about cathepsin K in oral tongue squamous cell carcinoma (OTSCC). Our aim was to describe the expression of cathepsin K in invasive OTSCC *in vitro* and in a series of clinical cancer specimens.

**Materials and Methods:**

OTSCC invasion *in vitro* was studied using invasive HSC-3 tongue carcinoma cells in 3D organotypic models. In total, 121 mobile tongue OTSCCs and 10 lymph node metastases were analyzed for cathepsin K expression. The association between cathepsin K expression and clinicopathological factors was evaluated.

**Results:**

Cysteine protease inhibitor E64 and cathepsin K silencing significantly (p<0.0001) reduced HSC-3 cell invasion in the 3D models. Cathepsin K was expressed in a majority of carcinoma and metastatic cells, but the expression pattern in carcinoma cells did not correlate with clinical parameters. Instead, the weak expression of cathepsin K in the invasive TME front correlated with increased overall recurrence (p<0.05), and in early-stage tumors this pattern predicted both cancer recurrence and cancer-specific mortality (p<0.05 and p<0.005, respectively).

**Conclusions:**

Cathepsin K is expressed in OTSCC tissue in both carcinoma and TME cells. Although the diminished activity and expression in aggressive tongue HSC-3 cells reduced 3D invasion *in vitro*, the amount of cathepsin K in carcinoma cells was not associated with the outcome of cancer patients. Instead, cathepsin K in the invasive TME front seems to have a protective role in the complex progression of tongue cancer.

## Introduction

Proteases are an integral part of the maintenance of the mesodermal connective tissue system, playing a fundamental role in processes from early development to growth and homeostasis [1]–[10]. Accordingly, alterations in the structure and expression patterns of proteases underlie many human pathological processes including arthritis, osteoporosis, neurodegenerative disorders, cardiovascular diseases, and cancer [11]–[15]. In carcinomas, proteolytic processing is combined with enhanced cell motility and reduced cohesion. These allow tumor expansion where the malignant cells protrude through the underlying basement membrane [16]. Hence, modeling of the tumor microenvironment has a central role in influencing tumor fate.

Cathepsin K is a lysosomal cysteine protease first characterized in osteoclasts [17]. It has an important role in many other tissues, as well [13], [14], [18], [19]. In general, the expression of cathepsin K in normal tissue seems to be associated with the process of extracellular matrix turnover. Cathepsin K is largely absent in skin, whereas dermal fibroblasts of surgical scars present strong cytoplasmic cathepsin K expression [11]. This suggests that cathepsin K is important for modeling of the dermal extracellular matrix. Accordingly, Codriansky *et al*. [20] have shown that cathepsin K is indeed a key factor in skin homeostasis, in which a decline in cathepsin K response in UVA-damaged fibroblasts results in an accumulation of abnormal elastin in the extracellular space. This confirms that cathepsin K is essential to the dynamic equilibrium between live mesenchymal cells and matrix synthesis and degradation.

Cathepsin K has been shown to have a significant role in many diseases, such as osteoarthritis and rheumatoid arthritis [21], atherosclerosis [22], the progression of cerebral aneurysms [12], and cancer progression [14]. Expression of cathepsin K has been demonstrated in malignancies including primary prostate and breast cancer [23], [24], both of which have a high propensity to metastasize to bone tissue. In breast cancer bone metastasis, inhibition of cathepsin K has dramatically reduced metastatic lesions [25]. In cutaneous squamous cell carcinoma, cathepsin K in stromal tissue facilitates cancer invasion [13], [18]. Cathepsin K expression has also been reported in epithelial cells of breast tumors and melanomas [14], [26].

The importance of cysteine proteinase cathepsins in the progress of oral carcinomas is relatively unknown [27]. In oral tissues and in oral squamous cell carcinomas (OSCCs), the presence of cathepsin K expression has not yet been described in the literature. Therefore, the aim of this study was to define the role of cysteine protease cathepsin K in the invasive process of OTSCC.

## Results

### Cathepsin Inhibitor E64 Reduced HSC-3 Invasion in the Myoma Organotypic Model

**Figure 1:**
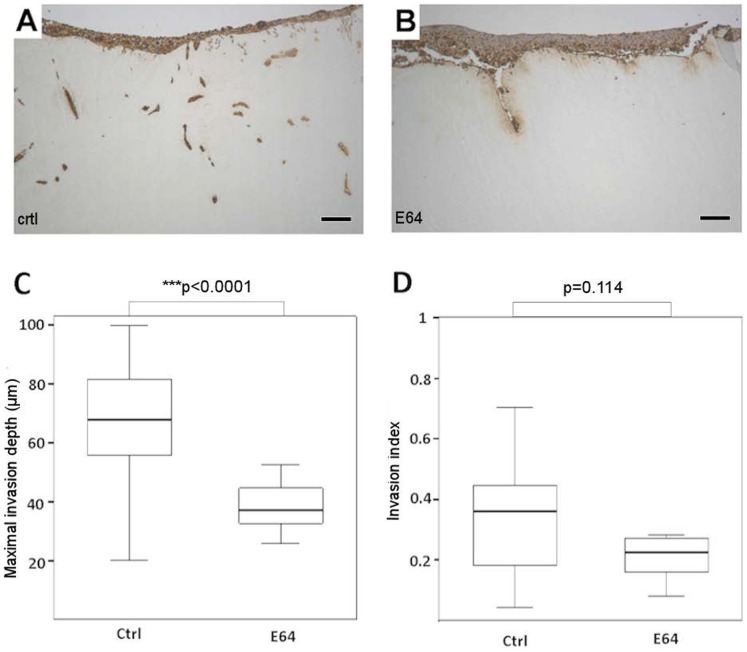
Cathepsin inhibitor E64 reduces HSC-3 tongue carcinoma cell invasion in the myoma organotypic invasion model. The effect of cathepsin inhibitor on the invasion of HSC-3 cells in the myoma organotypic assay was studied. E64 at 10 µM could prevent HSC-3 invasion as compared with the controls (A–B). Overall HSC-3 cell invasion depth (C) as well as invasion index (the ratio between the invaded area and the total area of AE1/AE3-stained HSC-3 cells) (D) were quantified. The invasion depth was significantly reduced (***p<0.0001), but the invasion index shows no significant reduction (p = 0.114). Scale bars 200 µm.

In order to evaluate the effect of broad spectrum cathepsin protease inhibitor (E64) on the invasion of aggressive tongue carcinoma cells, HSC-3 cells were grown in a myoma organotypic invasion assay (Figure 1). Interestingly, E64 was able to significantly prevent HSC-3 cell invasion in the myoma as compared with untreated cells (Figure 1A–1C) (*p*<0.0001). The invasion index (the ratio between the invaded area and the total area of AE1/AE3-stained HSC-3 cells) was also reduced, although the difference was not statistically significant (Figure 1D) (*p* = 0.114). In view of these results we have chosen to concentrate our efforts here on cathepsin K only, since it has already been described as a potent collagenolytic enzyme that is overexpressed in several malignancies [18], [23], [24], [28].

### Cathepsin K Knockdown Impairs HSC-3 Cell Invasiveness in the Myoma Organotypic Model

**Figure 2:**
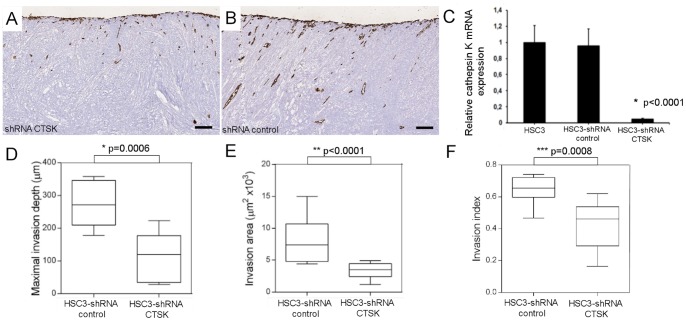
Specific cathepsin K knockdown significantly reduces HSC-3 cell invasion in the myoma organotypic invasion model. The knockdown of cathepsin K mRNA has significantly decreased the invasion of HSC-3 shRNA CTSK cells in the myoma organotypic model when compared with HSC-3 shRNA control cells (A–B). HSC-3 shRNA CTSK cells showed a reduction in cathepsin K mRNA levels when compared with wild type cells and HSC-3 cells transfected with a control shRNA (scramble) *p<0.0001 (C). The invasion depth, invasion area, and invasion index were all significantly reduced for HSC-3-shRNA CTSK cells (*p = 0.0006, **p<0.0001, and ***p = 0.0008, respectively) (D–F). Scale bars 200 µm.

HSC-3 cells knocked down for cathepsin K (HSC-3 shRNA CTSK) showed a significantly reduced amount of cathepsin K mRNA (p<0.0001) (Figure 2 C) and presented notably reduced invasion when compared with their wild type counterparts (Figure 2A–2B). Invasion depth (p = 0.0006), invasion area (p<0.0001), and invasion index (p = 0.0008) were significantly reduced in HSC-3 shRNA CTSK compared with control vector cells grown in the myoma discs (Figure 2D–2F).

### Cathepsin K is Expressed by HCS-3 and Stromal Cells in Organotypic Models

**Figure 3:**
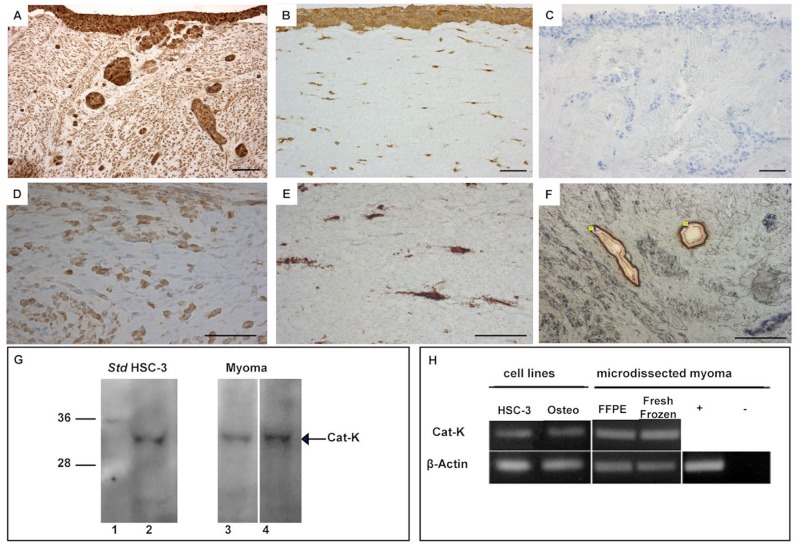
Cathepsin K expression in the myoma organotypic model. Invasive HSC-3 cells grown on myoma show intensive cathepsin K immunohistological staining (A). HSC-3 cells grown in type I collagen organotypic culture discs with embedded fibroblasts show cathepsin K staining in all cells present (B). Myoma tissue (without HSC-3 cells) as well as fibroblasts embedded in the collagen gel also stained with cathepsin K antibody (D–E). A negative control for immunostaining is shown (C). A Western blot confirmed that the monolayer cultures of HSC-3 cells (G, lane 2) and two distinct myoma tissue samples (without added carcinoma cells) contained cathepsin K (G, lanes 3 and 4). HSC-3 cells microdissected from the organotypic myoma model (F) of both formalin-fixed paraffin-embedded blocks (FFPE) and OCT-embedded frozen blocks (fresh frozen), as well as HSC-3 cells cultured *in vitro* in monolayers, express cathepsin K mRNA, as detected by RT-PCR (H). A differentiated human osteoclast progenitor cell line (Osteo) was used as a positive control for cathepsin K mRNA expression, represented by (+). Negative controls, where no sample was used, are demonstrated by (−) Scale bars 200 µm.

We wanted to verify the expression of cathepsin K mRNA and protein in tongue carcinoma HSC-3 cells cultured in two different types of 3D organotypic models. In addition to the human-tissue-derived myoma model used above, we tested the classic *in vitro* invasion assay using rat type I collagen discs embedded with human gingival fibroblasts [29]. Through immunohistochemistry we could demonstrate that HSC-3 cells expressed cathepsin K in both models (Figure 3A–3B). However, the myoma tissue, in the absence of invading carcinoma cells, also had detectable levels of cathepsin K immunoreactivity (Figure 3D), as did the fibroblasts embedded in the collagen gel (Figure 3E). Western blotting confirmed that the cultured monolayers of HSC-3 cells (Figure 3G, lane 2), and also the myoma tissue expressed cathepsin K, as demonstrated in two distinct myoma tissue samples (without HSC-3 cells) (Figure 3G lanes 3 and 4). To confirm specific cathepsin K mRNA expression by HSC-3 cells, we used laser microdissection to isolate the invading HSC-3 cells in the myoma tissue (Figure 3F) and by RT-PCR we revealed that the invasive HSC-3 cells contained cathepsin K mRNA (Figure 3H), confirming the expression of cathepsin K by oral tongue HSC-3 cells.

### Immunohistological Location of Cathepsin K in OTSCC Samples

**Figure 4:**
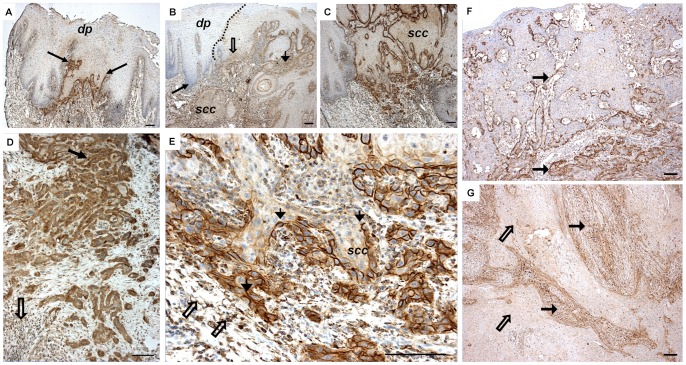
Cathepsin K immunostaining in invasive tongue cancer tissues and dysplastic oral epithelium. Cathepsin K in OTSCC tumors is localized in a few areas of dysplastic epithelium (dp) surrounding the cancer tissue (SCC) (A–B). A no staining area within a tumor slide with a score of (0) is shown by the first arrow (B). Other arrows, from left to right, show weak epithelial staining (+) and moderate stromal staining (++) (B). Cathepsin K shows a membranous, defined staining pattern in invading OTSCC carcinoma cells (C). The top arrow indicates an area with strong (+++) epithelial diffuse staining and moderate (++) stromal staining and the arrow below indicates stromal staining at the invasive front (D). At the invasive front, carcinoma cells show a cytoplasmic diffuse staining pattern. Big arrows indicate strong (+++) epithelial membranous staining. Empty arrows highlight individual cells in the stroma with moderate staining (++) (E). Arrows indicate different intensities of cathepsin K staining in superficial and invasive areas of tumor from weak (+) to moderate (++), illustrating the expression gradient observed in the samples (F). Example of strong stromal staining (full arrows) (+++) when epithelial staining is only moderate or weak (empty arrows) (G). Scale bars 200 µm.

In our 121 OTSCC patient samples, cathepsin K was detected in the great majority of cancers (only 4 cases were negative), including a few dysplastic areas surrounding the carcinoma tissue, as well (Figure 4A–4C). We could not detect cathepsin K in the morphologically normal-looking epithelium of the tongue (not shown). In carcinomas, cathepsin K was present in both carcinoma and stromal cells. Interestingly, the carcinoma cells showed two kinds of staining patterns: a localized (membranous) and a diffuse (cytoplasmic) distribution (Figure 4D–4E). The membranous staining pattern was usually visible in the most superficial to middle areas of the tumor, being gradually replaced by the cytoplasmic type.

### Cathepsin K Expression is Distinct in Epithelial and Stromal Cells

**Figure 5:**
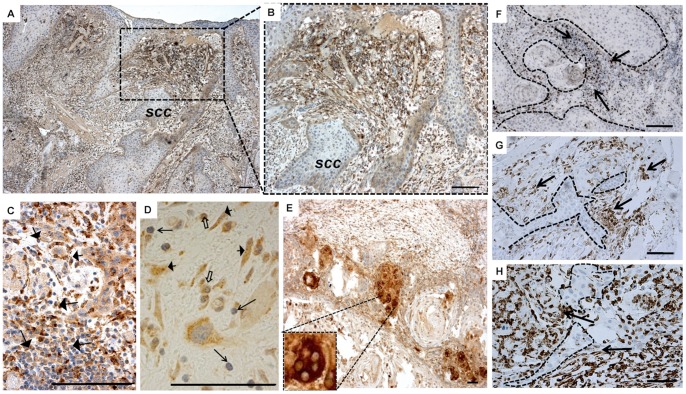
Cathepsin K is expressed by various stromal cells. Adjacent stroma of some OTSCC tumors shows strong staining for cathepsin K (A–B). Arrows show several round nuclear inflammatory and spindle mesenchymal cells in OTSCC stroma that have abundant cathepsin K staining (C–D). In matching areas of the TME, where cathepsin K was expressed, we also observed cells positive for the selective immunohistochemical markers CD45 (F), CD68 (G), and vimentin (H), confirming the presence of inflammatory and mesenchymal cells. Arrows indicate positive cells and dotted lines indicate OTSCC cell islands (F–H). Multinucleated giant tumor cells are intensively stained for cathepsin K (E). Scale bars 200 µm.

In some samples, we observed a gradient of staining intensity accompanying the variations in expression of cathepsin K. This seems to be an epithelial-to-mesenchymal pattern shift in the expression of cathepsin K as the tumor begins to reach more invasive areas of the tumoral tissue. Also, even when there is no gradient in expression from superficial to deeper areas of the tumor, the intensity of the expression of cathepsin K by the stroma and epithelial cells seemed to be inversely correlated, with tumor cells presenting higher cathepsin K expression in areas where stromal staining was weaker, and *vice versa* (Figure 4F–4G). The expression of cathepsin K by TME cells was observed as abundant diffuse staining in various inflammatory and spindle-shaped cells (Figure 5A–5D). Staining with selective markers CD45 (for cells of hematopoietic lineage), CD68 (for cells of the monocyte/macrophage lineage), and vimentin (for cells of mesenchymal origin) was used to confirm the presence of inflammatory and mesenchymal cells at the matching location with cathepsin K in the TME (Figures 5F–5H). The cathepsin-K-positive cells at the stroma were present both in the most superficial areas (Figures 5A–5B) and surrounding the invasive front (Figures 5C–5D). Some carcinomas contained multinucleated giant cells, which always stained intensively for cathepsin K (Figure 5E), similar to osteoclasts of the bone (not shown).

### Cathepsin K Expression Pattern is an Independent Predictor of Disease Recurrence and Patient Mortality

**Figure 6:**
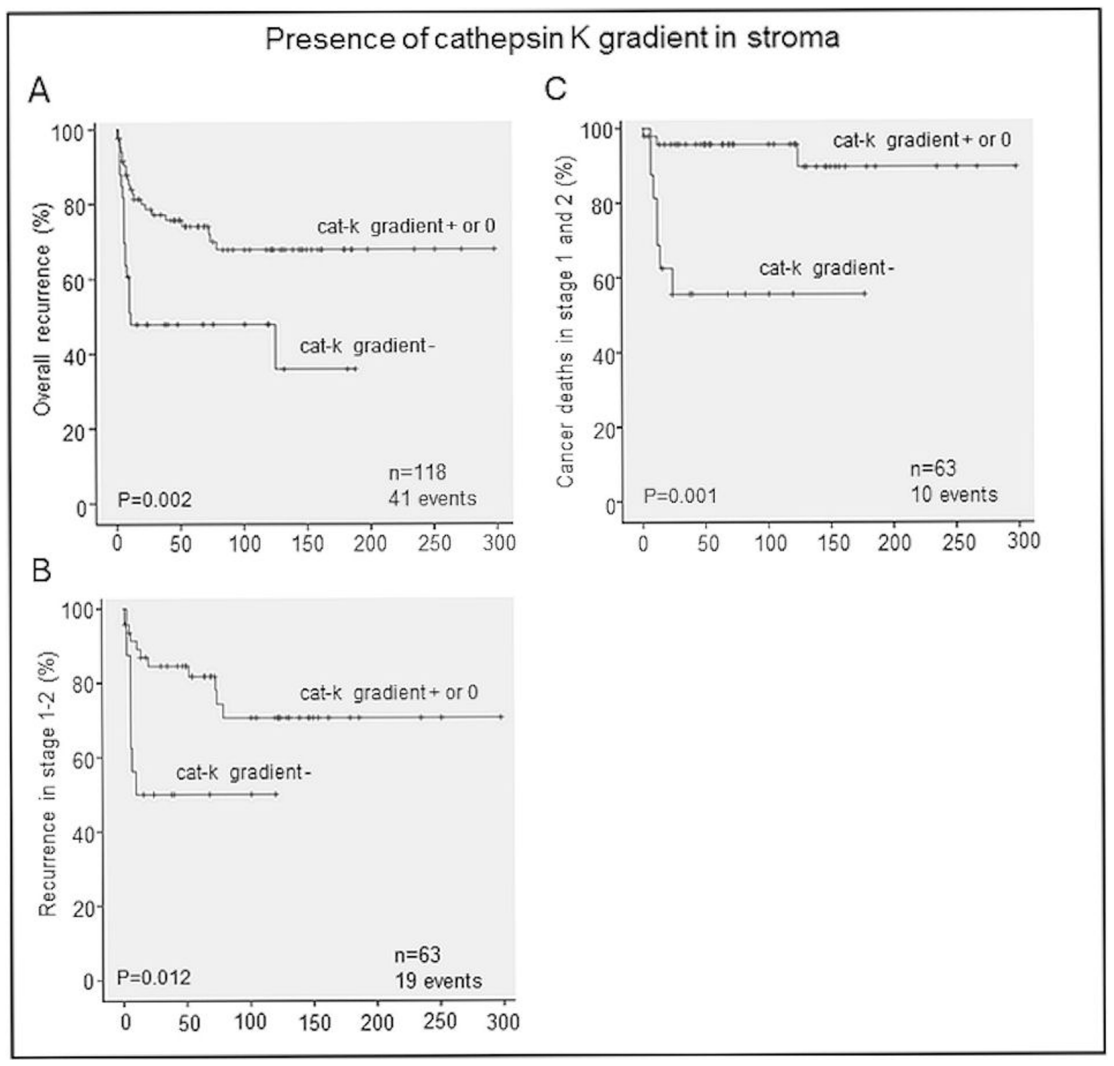
Weaker staining of cathepsin K in the invasive front correlates with shorter recurrence-free survival and increased mortality. Survival analyses show a shorter overall recurrence-free interval in patients with a lower cathepsin K staining gradient (cat-K gradient −) compared with those with either higher (cat-K gradient +) or no gradient (gradient 0) of cathepsin K staining intensities in the invasive front compared with the upper part of the tumor (A), especially seen in patients with stage 1 or 2 OTSCC (B). Disease-specific survival of patients with stages 1 and 2 OTSCC tumors was better in patients with a positive or no gradient of cathepsin K staining compared with those with a negative gradient (C).

We correlated our immunohistopathology analyses of cathepsin K expression and compared them with patient clinicopathological data. In our univariate analyses, there were no correlations between our parameters and gender, age, grade, stage, lymph node status, or therapy. The only correlation found was between a gradient towards diminished expression of cathepsin K in the TME at the invasive front and increased overall recurrence (*p*<0.05). This correlation was confirmed in our survival studies (Figure 6A). There was also a correlation between more recurrences and cancer-specific deaths in stages 1 and 2 (Figure 6B–6C). In our multivariate analyses, we confirmed that weaker expression of cathepsin K in the invasive TME front was an independent predictor of overall recurrence (HR 2.594, 95% CI [1.394–4.827]). In early-stage tongue cancers, the aforementioned cathepsin K expression pattern predicted recurrence (HR 3.093, 95% CI [1.214–7.877]) and cancer-specific mortality (HR 9.306 95% CI [2.303–37.609]).

### Cathepsin K Expression Pattern is Similar in Primary Tumors and Lymph Node OTSCC Metastases

**Figure 7:**
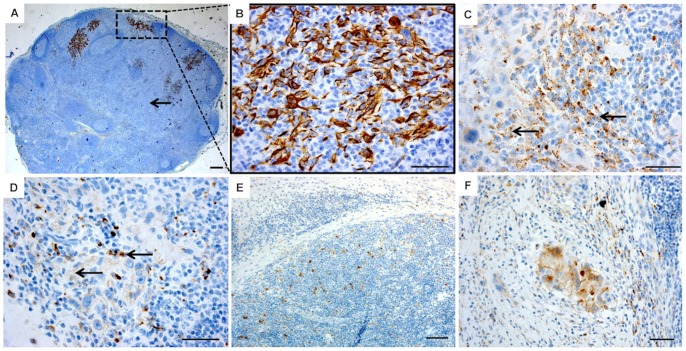
Cathepsin K immunostaining pattern in lymph node metastases of OTSCC is similar to the primary tumor. Metastatic carcinoma cells were identified with pancytokeratin staining (A–B), followed by cathepsin K staining of the paired primary OTSCC (C) and lymph node metastases (D) (n = 10, the paired sections were taken from the same patient). The arrows indicate similar cathepsin K staining patterns in both the primary tumor and in the lymph node metastases. Lymph nodes without metastases contain some cathepsin K positive cells (E), but the stromal reaction drastically changes in the presence of cathepsin K positive OTSCC cells (F). Scale bars 200 µm.

In the ten primary cancer cases with corresponding lymph node metastases analyzed (Figure 7A–7C), we observed that metastatic OTSCC cells in lymph nodes were also positive for cathepsin K (Figure 7D–7F). Not only that, the organization of the stroma seemed to mimic the one found at the primary tumor, with inflammatory cells organized around OTSCC cells and expressing increased amounts of cathepsin K (Figure 7D). The vast majority of metastasis-free lymph nodes did not express cathepsin K, with only a very few positive randomly deposited cells (Figure 7E).

## Discussion

Previously, cathepsin K expression in cancer was thought to be limited to osteoclasts, having distinct but synergistic effects on matrix metalloproteases (MMPs) in bone resorption associated with tumor metastasis [17], [30]. Its presence has also been demonstrated in cutaneous SCC tumor stroma [13], [14], [18], [19], and it seems to be expressed to a higher degree in invasive tumors when compared with breast and melanoma *in situ* carcinomas [14], [18], [26]. Cathepsin K activity is complex, with roles in angiogenesis, bone remodeling, and progenitor cell mobilization. With these important roles in tissue homeostasis, it is interesting to note that its expression in primary tumors, including OTSCCs, still has not been thoroughly investigated. Much less is known about its participation in the modulation of other molecules necessary for local tumor invasion [18], [26]. We showed here, for the first time, that cathepsin K is abundantly expressed by OTSCC cells in addition to the tumor microenvironment (TME), and that its expression pattern by tumor cells has prognostic significance.

As first demonstrated in our myoma organotypic experiments, the invasiveness of HSC-3 cells was markedly reduced with the introduction of cathepsin inhibitor E64, pointing to an important role for tumor cell cathepsins. The choice of studying cathepsin K more closely was likely to yield interesting results, since it was already very well described in several studies that demonstrate the power and enhanced activity of cathepsin K in the TME [18], [23], [24], [28]. With our specific knockdown of cathepsin K mRNA translation, we have established that although invasion was not completely abolished in the myoma organotypic model, it was significantly reduced–showing that cathepsin K is a powerful driver of HSC-3 OTSCC cell invasiveness. In our OTSCC patient samples, cathepsin K expression in the stromal component of OTSCCs surprisingly seems to be protective, since a poorer prognosis for stages 1 and 2 was strongly correlated to weaker cathepsin K expression at the invasive TME front. In this environment, regulation of the expression of cathepsin K between stroma and carcinoma cells appears to be far more complex and dynamic, where cathepsin K expression intensity is complementary between stromal and epithelial cells. We postulate that cathepsin K expression may lead to a different prognosis depending on whether it is expressed more in the OTSCC cells or by the cells in the surrounding stroma.

Other reports have explored in more detail the complex nature of cathepsin K regulation and the crosstalk between the TME, mainly carcinoma-associated fibroblasts (CAFs) [31], where cooperation between stromal and epithelial tumor cells has promoted the expression of cathepsin K, and enhanced tumor invasive capacity. This highlights the importance of the TME in the behavior of these cells, since expression intensity outside the tumor front was not considered to be of prognostic significance. In lymph node metastases, the pattern of cathepsin K expression was similar to the one found in the corresponding primary tumors, indicating that invasive tumor cells quickly mobilize the host stromal cells and modify their new surroundings. Therefore, this indicates that the complexity of the TME seems to be more significant than the individual tumor elements [14], [18].

In our patient samples, cathepsin K was present in OTSCC tumor epithelial cells, but not in normal mucosa keratinocytes. This is in line with previous findings where cathepsin K was occasionally expressed by skin keratinocytes only in young surgical scars, and focally in epithelial cells of the breast [14]. However, in skin squamous cell carcinomas, epithelial expression of cathepsin K was reported absent [13] or focal and weak compared with the TME [26]. In our results, the TME and carcinoma cells of OTSCC presented significant, but fluctuating expression of cathepsin K. We believe that our high number of cases and our focus on only OTSCC will give a better understanding of how the molecular differences between tumors of different locations might explain their often different clinical outcomes.

The reasons for the variable expression of cathepsin K in the invasive carcinoma cells and tumor front of the TME and its seemingly complementary relationship *in vitro* can obviously be due to the more complex regulation and interaction of cells in cancer. There are some reports in the literature that discuss cathepsin K in tumors [13], [14], [18], [23], [24], [26], but none on OTSCCs and their microenvironments. We can readily postulate two explanations for the observed inverse relationship between patient survival and cathepsin K expression in OTSCC stroma. One explanation is that lower cathepsin K expression leads to a diminished reduction in growth factor activity, which is already shown to be a plausible phenomenon during airway development, where TGF-β1 levels are affected [32]. Alternatively, we can presume that more enhanced cathepsin K activity exists in sites where it is needed to complement the absence of other enzymes. As the enzymatic activity grows in the stroma, there might be a feedback mechanism of active cathepsin K repression, such as activation of cystatins. In another approach, several enzymes overexpressed at the tumoral front, through some unspecific activity, might be miscleaving the pro-cathepsin K present in these tissues, therefore effectively halting their maturation process [33]. *In vivo,* the situation is far more complex, and the role of growth factors and feedback mechanisms can be more determinant in the final prognosis, which is strongly influenced by metastasis and growth capacity of the tumor.

Due to its potential role in osteoporosis, cathepsin K is currently one of the cysteine proteinases most intensively investigated by the pharmaceutical industry [34]. Inhibitors of cathepsin K have shown promising results in clinical trials for postmenopausal osteoporosis, indicating that these drugs may soon become commercially widely available [34], [35], [36]. However, since the function of cathepsin K is not yet fully clarified in OTSCC, and especially in the TME, our results suggest that we should be aware of the possible effects of cathepsin K inhibitor treatment on changes in the protective/destructive balance of cathepsin K expression in oral tongue tumors.

In conclusion, these results support the idea that oral carcinoma cells can use multiple mechanisms as they infiltrate into adjacent tissues, bringing another level of complexity to the TME. Based on this, cysteine proteases, such as cathepsin K, seem to be important regulators of OTSCC invasion. As our knowledge of cathepsin K participation in the development of tumors is expanding, there is also interest in evaluating its contribution to development and invasion in OTSCC, providing more tools for understanding, predicting, and developing better therapeutic alternatives for stemming OTSCC progression.

## Materials and Methods

### Ethics Statement

Patients signed an informed consent form and data inquiry was approved by the National Supervisory Authority for Welfare and Health (VALVIRA), # 6865/05.01.00.06/2010 (5.10.2010), and the Ethics Committee of the Northern Ostrobothnia Hospital District, statement 49/2010 (16.8.2010). Use of patient material for this study was approved by the Northern Ostrobothnia Hospital District Ethics Committee (statement #8/2006 and amendment 19/10/2006).

### Cell Culture

Human HSC-3 tongue carcinoma cells (JCRB Cell Bank 0623, National Institute of Health Sciences, were cultured in 1∶1 of DMEM and Ham’s F12 culture medium supplemented with 10% foetal bovine serum, 100 U/ml penicillin, streptomycin (100 µg/mL), 250 ng/mL Fungizone (Invitrogen, USA), 50 µg/mL ascorbic acid, and 0.4 ng/ml hydrocortisone, as described in detail earlier [37]. The gingival fibroblasts were cultured as described before [29].

### Osteoclast Progenitor Cells and Differentiation

Isolation of osteoclast progenitor cells from human bone marrow and human osteoclast differentiation were carried out as described before in [38]. Briefly, non-adherent mesenchymal progenitor cells were collected and the monocyte fraction was purified. Cells were plated on ultrasonicated human cortical bone slices for seven days; half of the culture medium was replaced twice a week with fresh medium (demi-depletion). After seven days, the pH of the culture medium was lowered to 6.8 with sterile 0.5 M HCl and culturing was continued for an additional three days, after which the cells were lysed for RNA isolation.

### Cathepsin K shRNA Transduced Cells

Target cells were plated in a 12-well plate to 50% confluence 24 hours prior to viral infection with 1 ml of complete medium. CTSK shRNA and control shRNA lentiviral particles (reference numbers sc-29936-V and sc-108080) were purchased from Santa Cruz Biotechnology (USA). We prepared fresh medium with 5 µg/ml of Polybrene™ and added 1 ml of this medium to cathepsin K shRNA and control shRNA lentiviral particles and incubated them overnight. We then split cells in a 1∶5 concentration and continued incubation for 48 hours in complete medium. Selection was achieved with Puromycin dihydrochloride (Sigma-Aldrich, USA). Resistant colonies were expanded for detection of stable shRNA expression through real-time PCR (qPCR).

### Collagen Gel Organotypic Culture

The 3D collagen gel experiment was done as described previously by Nurmenniemi *et al*. [29]. Briefly, 8 volumes of rat-tail-derived type I collagen (3.45 mg/ml; BD Biosciences, Bedford, MA), 1 volume of 10×DMEM (Sigma-Aldrich), and 1 volume of fetal bovine serum with gingival fibroblasts (final concentration in gel 7×10^5^ cells/ml) were mixed on ice and allowed to polymerize on 24-well plates at 37°C for 30 minutes. After polymerization, 7×10^5^ cancer cells were added on each gel. The next day the gels were detached from the well walls, allowed to contract for 4 hours, and lifted onto collagen-coated (BD Biosciences) nylon disks (Prinsal Oy, Finland) resting on curved steel grids (3×21×21 mm). The grids were placed on 6-well plates, and a sufficient volume of culture medium (2.5 ml) was added to reach the undersurface of the grid, generating an air-liquid interface. This was day 1 of the culture. The media of the organotypic cultures were changed every 3 days for 14 days, whereupon the cultured tissues were harvested.

### Human Myoma Organotypic Culture and Quantification of Invasion Results

The myoma organotypic experiment was done as described previously [29], with the following changes. Ten days prior to the experiment the myoma disks were equilibrated at +4°C in cell culture medium, which was changed regularly. The myoma disks were pre-incubated 12 h prior to the experiment in a culture medium containing cathepsin inhibitor E64 (Sigma-Aldrich) at a concentration of 10 µM and the HSC-3 cells for 1 hour at 37°C in a humidified 5% CO_2_ atmosphere. Then the myoma disks (3 per group) were placed into Transwell inserts and 7×10^5^ cells per myoma were added and allowed to attach overnight in media containing E64 (10 µM). Next, the myoma disks were moved to uncoated nylon disks on curved steel grids in 12-well plates with 1 ml of medium containing 10 µM E64. During the experiment, the medium with the inhibitor was changed every third day. The myoma cultures were maintained for 10 days and then the disks were fixed in 4% neutral-buffered formalin overnight. The myoma disks were bisected and either embedded in OCT compound and frozen at −70°C until use or dehydrated and embedded in paraffin. Paraffin blocks were cut into 5-µm sections and tissue sections were stained with primary antibody against pancytokeratin, clones AE1/AE3, at a 1∶250 dilution (Dako, Copenhagen, Denmark) to detect carcinoma cells of epithelial origin from the HSC-3 myoma tissue sections, and the depth of invasion and invasion index of the HSC-3 cells were quantified as described previously [29], [39].

### Patient Samples and Clinicopathological Data

Archival specimens of 121 OTSCC samples, surgically treated at the Oulu University Hospital in 1981–2009, were retrieved from the Oulu University Hospital, Department of Pathology. The median age of the patients was 65 years (range 27–99). The median follow-up time was 121 months (range 24–298 months) in the surviving patients (n = 54). The median follow-up time for the study patients was 53 months (range 1–298 months). Patient survival data were acquired from Statistics Finland and other relevant data from patient records (Table 1). We could not retrieve treatment data from three of the patients or survival data from two of the patients. We also used ten samples of lymph nodes with metastatic OTSCC lesions, which were analyzed only for their immunohistopathological features.

**Table 1:** Data retrieved from the files of patients whose samples were used in this study.

Patient clinical data			
		N	%
Age at diagnosis			
<55 yrs		40	33.0
55–70 yrs		33	27.3
>70 yrs		48	39.7
Sex			
Male		60	49.6
Female		61	50.4
Tumor grade			
1		44	36.4
2		62	51.2
3		15	12.4
Tumor stage			
1–2		64	52.9
3–4		57	47.1
Neck lymph nodes			
Negative		81	66.9
Positive		40	33.1
Recurrence			
No		80	66.9
Yes		41	33.1
Adjuvant therapy			
No		69	57.0
Radiotherapy		38	31.4
Radio- and chemotherapy		11	9.1
Missing		3	2.5
Total		121	

### Immunohistochemistry

For HSC-3 cells in the myoma and collagen discs as well as the OTSCC and lymph node samples, cathepsin K immunostainings were performed on paraffin-embedded sections. Specimens of OTSCC were previously selected to be representative of the tumor mass in the resected specimens. The slides were deparaffinized, rehydrated, and washed with 0.3% hydrogen peroxide to inhibit endogenous peroxidase. After a high-temperature antigen retrieval procedure in Tris-EDTA buffer for 15 minutes, the slides were washed with PBS and incubated with cathepsin K primary antibody (Biovendor, USA), clone: 3F9 for 1 h at room temperature at a 1∶500 dilution. For OTSCC clinical samples we also employed selective markers for cells of monocyte/macrophage lineage (CD68, clone: PG-M1 at a 1∶10000 dilution, Dako), cells of hematopoietic lineage (CD45, clone: A20 at a 1∶400 dilution, BioLegend, USA), and cells of mesenchymal origin (vimentin, clone: V9 at a 1∶1500 dilution, Dako), also for 1 h at room temperature. For lymph nodes, also primary antibody against pancytokeratin, to detect metastatic carcinoma cells (clones: AE1/AE3, at a 1∶250 dilution, Dako), was used. To detect antibody reactions, we used a Dako Envision kit (Dako) with Diaminiobenzidine (Dako basic DAB-kit) as a chromogen. Counterstaining was done in a Dako Autostainer (Dako). Validation of our immunohistochemical analysis was done using positive controls (human bone tissue) and two series of negative controls (by omitting the primary antibody and by replacing the primary antibody with mouse primary antibody isotype control).

### Western Blot

Total protein from cultured HSC-3 and myoma tissue samples (without invading HSC-3 cells) was extracted by homogenizing the cells and tissue in a lysis buffer (50 mM Tris, 10 mM CaCl_2_, 150 mM NaCl, 0.05% Brij, pH 7.5) on ice, rotating them at 4°C for 4 h and collecting the supernatant by centrifugation at 12000×g for 10 min. Western blot analysis using polyclonal cathepsin K in a 1∶50 dilution (Abcam, Cambridge, UK) was done as described previously [40].

### Laser Capture Microdissection

Myoma tissue samples (with invading HSC-3 cells) were fixed in buffered formalin and embedded in paraffin, or snap-frozen in OCT compound (Sakura, USA). Sections were cut and mounted on polyethylene naphthalate (PEN) membrane-coated slides (P.A.L.M. Microlaser Technologies, Germany). Areas of invading HSC-3 cells were cut and collected by pressure catapulting from the myoma tissue by means of laser microdissection using the P.A.L.M. Robot-microlaser system (P.A.L.M. Microlaser Technologies) according to the manufacturer’s instructions.

### Cathepsin K RT-PCR and qPCR

RNA from HSC-3 cells, HSC-3 shRNA CTSK, and shRNA control, as well as from differentiated human osteoclast progenitor cells and microdissected samples was isolated with TRIzol reagent (Invitrogen) or with an RNeasy FFPE kit (QIAGEN, USA) according to the manufacturer’s protocol. For the first strand cDNA synthesis, 1 µg of total RNA per sample was used and the reaction was performed with a superscript enzyme (RevertAid™ Reverse Transcriptase enzyme, Fermentas-Thermo Scientific, USA). We used cathepsin K primers (forward 5′-ccgcagtaatgacacccttt-3′ and reverse 5′-gcacccacagagctaaaagc-3′) and beta-actin (forward 5′-aactgggacgacatggagaaaa-3′ and reverse 5′-ag aggcgtacagggatagcaca-3′) as a reference gene. Briefly, PCR reactions were done in 45 cycles of 5 minutes at 95°C, 2 minutes at 94°C, 1 minute at 54°C, 30 seconds at 72°C, and a final extension at 72°C for 10 minutes, using DNA polymerase AmpliTaqGold™ (Fermentas). The PCR products were then analyzed in a 1% agarose gel and stained with ethidium bromide. The size of the amplification products was confirmed with a 100 bp ladder commercial molecular weight marker (Fermentas). For qRT-PCR we used the StepOnePlus Real Time PCR System (Applied Biosystems Inc., USA). Each experiment was done with 12.5 µl of SYBR® Green PCR Master Mix (Applied Biosystems Inc., USA), 2 µl of 10 nM of each primer, and 1 µl of sample. β-actin was used as the internal control. All the experiments were repeated three times.

### Immunohistochemical Assessment of Cathepsin K Expression

Cathepsin K immunostaining was analyzed by three independent researchers (C.C.B., J.H.K., and T.S.) who had no previous knowledge of the clinical data. Expression of cathepsin K was evaluated using an intensity-of-labeling scale (no staining = 0, weak staining = +, moderate staining = ++ and strong staining = +++) in overall tumor epithelial and overall as well as invasive stromal staining. The process of scoring the intensity of staining was completed first by calibrating the researchers with cases of clear examples of each of the above-mentioned classification tiers (examples in Figure 4). After consensus, the analysis of each slide started with a no staining area with a score of (0). Following that, we analyzed the following criteria in the same order: overall stroma staining intensity (0 to +++), overall epithelial staining intensity (0 to +++), and overall invasive stroma staining (0 to +++). In all cases we selected 3 different areas for each parameter and only considered scoring if consensus was reached. Other parameters considered were the pattern of cathepsin K expression in superficial and invasive front tumor epithelia (membranous or diffuse staining) and cathepsin K expression in an invasive front compared with superficial tumor stroma (more, less, and no difference) (Figures 4 and 5).

### Statistical Analysis

Differences in tumor metastasis, size, and sample staining between groups were compared with the Mann-Whitney or Student’s T-test. To estimate statistical significance in the myoma samples, an independent sample T-test was performed. Results with *p*<0.05 were considered statistically significant. *In vitro* invasion data are expressed as box blots. The line across the box indicates the median; the box contains the values between the 25th and 75th percentiles, and the whiskers show the highest and lowest values. We used PASW Statistics 18 (IBM corp.) software for statistical analyses of the patient material. A chi-square test was used to calculate statistically significant differences between prognostic and clinicopathological variables. Survival tables were calculated according to the Kaplan-Meier method, and were compared with the log-rank test. Multivariate survival and recurrence analyses were done with the Cox proportional hazards model using the following covariates: gender (male or female), age at the time of diagnosis (<55 yrs, 55–70 yrs, and >70 yrs), tumor stage (1–2 and 3–4) and tumor histologic grade (1, 2, and 3). For stage 1–2 tumors, only gender, age, and grade were used. Cox regression was done using backward stepwise selection of variables, and a *p* value of 0.05 was adopted as the limit for inclusion of a covariate. The backward stepwise algorithm was used to pick the best combination of prognostic factors to explain mortality or recurrence in the study population. The hazard ratios and 95% confidence intervals are provided for each covariate.
